# Nucleotide sequence as key determinant driving insertions at influenza A virus hemagglutinin cleavage sites

**DOI:** 10.1038/s44298-024-00029-1

**Published:** 2024-05-13

**Authors:** Monique I. Spronken, Mathis Funk, Alexander P. Gultyaev, Anja C. M. de Bruin, Ron A. M. Fouchier, Mathilde Richard

**Affiliations:** 1 Department of Viroscience, Erasmus Medical Centre, Rotterdam, The Netherlands; 2 Institute for Biochemistry & Research Center for Emerging Infections and Zoonoses (RIZ), University of Veterinary Medicine Hannover, Hannover, Germany

**Keywords:** Virology, Influenza virus

## Abstract

Highly pathogenic avian influenza viruses (HPAIVs) emerge from H5 and H7 low pathogenic avian influenza viruses (LPAIVs), most frequently upon insertions of nucleotides coding for basic amino acids at the cleavage site (CS) of the hemagglutinin (HA). The exact molecular mechanism(s) underlying this genetic change and reasons underlying the restriction to H5 and H7 viruses remain unknown. Here, we developed a novel experimental system based on frame repair through insertions or deletions (indels) of HAs with single nucleotide deletions. Indels were readily detected in a consensus H5 LPAIV CS at low frequency, which was increased upon the introduction of only one substitution leading to a longer stretch of adenines at the CS. In contrast, we only detected indels in H6 when multiple nucleotide substitutions were introduced. These data show that nucleotide sequence is a key determinant of insertions in the HA CS, and reveal novel insights about the subtype-specificity of HPAIV emergence.

## Introduction

Wild aquatic birds are the original reservoir of influenza A viruses, which are categorised by the antigenic properties of their hemagglutinin (HA) and neuraminidase (NA) surface glycoproteins. To date, 17 HA and nine NA subtypes have been detected in wild aquatic birds^1,2^. Influenza A viruses generally cause asymptomatic or mild infections in birds, and are referred to as low pathogenic avian influenza viruses (LPAIVs)^3^. Upon the introduction of LPAIVs of the H5 and H7 subtypes in terrestrial poultry, highly pathogenic avian influenza viruses (HPAIVs) may emerge. HPAIVs cause severe disease in poultry with mortality rates as high as 100%^4^. Besides these disastrous consequences on animal welfare and the poultry industry, spill-over events to humans pose a continuous pandemic threat^5^.

Post-translational cleavage of HA by host cell proteases is necessary for HA to be fusogenic and the virus to be infectious^1^. The HA proteins of LPAIVs have a monobasic cleavage site (CS) that is cleaved by trypsin-like proteases, predominantly expressed in the respiratory and intestinal tracts of birds^6,7^. In the vast majority of cases, the conversion from LPAIV to HPAIV is the result of the insertion of nucleotides coding for basic amino acids at the HA0 precursor protein CS, leading to a multi-basic cleavage site (MBCS)^8,9^. MBCSs are cleaved by furin-like proteases that are ubiquitously expressed, supporting systemic virus dissemination and severe disease in gallinaceous species^10^. Decades ago, different mechanisms of MBCS acquisition were identified upon the analysis of MBCS sequences of HPAIVs: (i) nucleotide substitutions and (ii) nucleotide insertions possibly due to either stuttering and/or backtracking of the influenza virus RNA-dependent RNA polymerase (RdRp) or, (iii) in the case of some H7 viruses, to non-homologous recombination with exogenous viral or host RNA^11–13^. The exact molecular mechanisms underlying MBCS acquisition via insertions remain unknown to date. Additionally, it remains unknown why HPAIVs have so far only evolved from H5 and H7 LPAIV precursors. Artificial introduction of an MBCS into non-H5/H7 HAs generally led to trypsin-independency in vitro^14–17^, indicating that the absence of an MBCS in naturally occurring non-H5/H7 viruses is not due to incompatibility at the protein level. The restriction of HPAIVs to H5 and H7 subtypes might therefore be due to differences and/or constraints at the RNA level. RNA structures have been suggested to play a role in insertion generation at the HA CS^11,18–20^. Subtype-specific conserved secondary RNA stem-loop structures have been predicted at the HA CS^19,21^. However, RNA structure predictions of HAs from subtypes other than H5 and H7 have identified similar putative structures, suggesting that RNA structures might not be the only important factor that contributes to insertions in HA CSs^21^. The H5 and H7 CS sequences have been shown to stand out by their high purine content, suggesting that specific sequences and codon composition^11,18,20,22–25^ may be important for MBCS acquisition and subtype restriction of HPAIV genesis^25^.

Research aimed to study the drivers of MBCS acquisition has been hampered by the fact that it is difficult to mimic this process in a laboratory setting, both in vitro and in vivo. H5 and H7 LPAIVs converted in rare occasions to HPAIVs and only when selection pressure for trypsin independency was applied^26–31^, extensive passaging was performed^26,29,30,32,33^, or non-basic amino acids at the CS were substituted to basic ones^22–24,34–36^. Some of these experiments were performed with virus isolates instead of clonal viruses, making the distinction between de novo insertions and the presence of viral quasispecies with mutated CSs impossible^26–33^. Other shortcomings were the low throughput and sensitivity as viruses with mutated CSs have to outcompete the wild-type (WT) virus in order to be reliably detected. To cope with these limitations, we developed a sensitive system with which indels at the HA CS can be readily detected. Single nucleotide deletions (SNDs) were introduced at the HA CS, leading to a non-functional HA protein. Upon use of these templates in reverse genetics, viruses with HAs that contain indels restoring the reading frame are under strong selective advantage without competition with a WT virus. Using this experimental system, we investigated the impact of nucleotide sequence at the HA CS on insertions and deletions (indels), in order to shed light on subtype-restriction of LPAIV to HPAIV conversion. We showed that nucleotide sequence was important in the acquisition of indels in the H5 and H6 CS region. Indels in the H5 CS were easily facilitated by a one nucleotide substitution increasing the length of the adenine/uracil (A/U)-stretch while indels in the H6 CS were only detected upon five or six nucleotide substitutions.

## Results

### Indels were readily detected at the H5 CS

We aimed to develop a novel experimental system with which insertions at the HA CS can be detected with a high sensitivity. SNDs were introduced, leading to a (−1) frameshift in the HA reading frame and thus changing the coding sequence. Upon use of these templates in reverse genetics, functional virus can only be produced if the frame of the HA protein is repaired by indels. This system ensures that rare indels that would not necessarily confer a selective advantage over the WT virus can be detected.

**Fig. 1:**
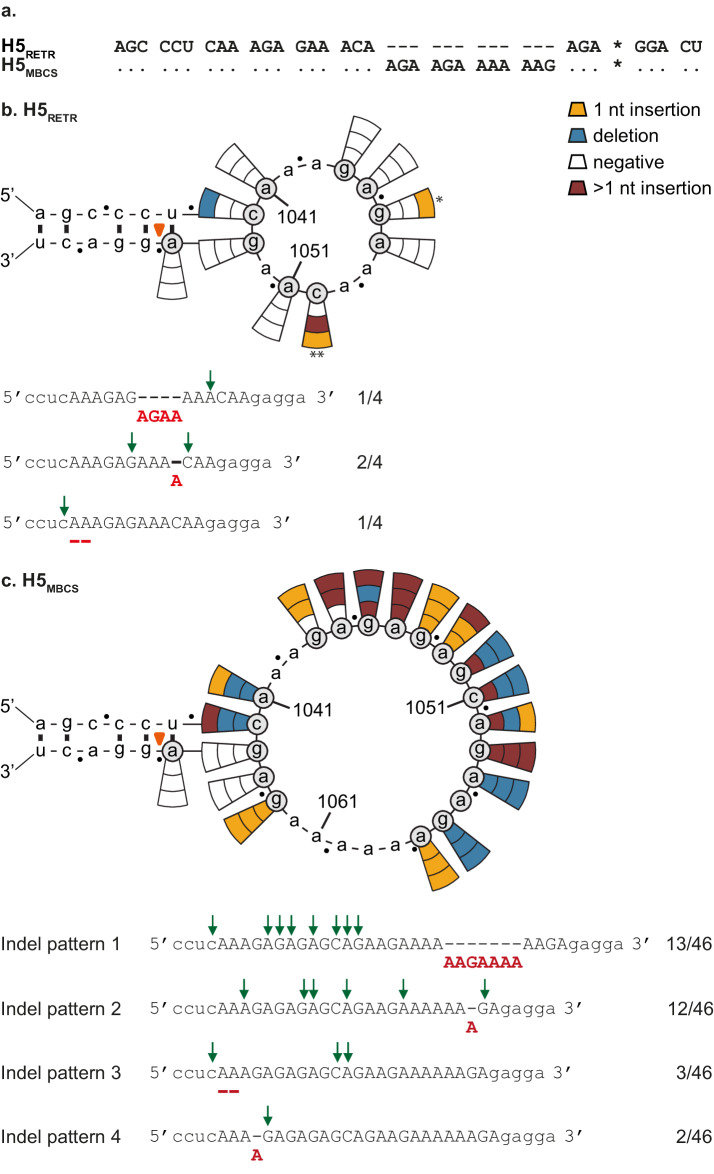
Higher indel detection was observed in the H5_MBCS_ CS than in that of H5_RETR_. The predicted RNA stem-loops serve as a basis to schematically represent the data. The black dots delineate codons. The orange arrow head indicates the start of the codon of the HA2 N-terminal glycine. Grey circles indicate the SNDs that were generated. Connecting pie slices show the results of three independent reverse genetics experiments, with the colour of the wedge indicating the nature of the observed indel, according to the legend at the top. The asterisks indicate results that are shown in multiple figures, with the number of asterisks identifying a given result. Observed indel patterns and corresponding frequencies are indicated below the structures, the other detected indels are shown in Supplementary Table 1a. The sequence of the loop and stem of the predicted RNA structures are shown with capital and lowercase letters, respectively. The green arrows indicate the location of each SND present in the HAs in which the corresponding indel pattern was detected. Sequences in all figures are indicated in cRNA positive sense orientation. **a** Alignment of H5_RETR_ and H5_MBCS_ CS sequences shown in Fig. 1. The asterisk indicates the border between HA1 and HA2. Results from testing (**b**) H5_RETR_ HA_SND_s and (**c**) H5_MBCS_ HA_SND_s.

In order to study indels in an H5 with a LPAIV CS, the CS of the H5 A/Indonesia/5/2005 (A/Indo/5/05) HPAIV HA was mutated to match the H5 LPAIV amino acid and nucleotide consensus P4 to P1 RETR sequence (H5_RETR_)^25^ (Supplementary Fig. 1, Fig. 1a). SNDs were introduced into the H5_RETR_ CS and the resulting plasmids were co-transfected with the remaining seven reverse genetics plasmids of A/Indo/5/05 as virus backbone. Each HA with a SND (HA_SND_) was tested in three independent reverse genetics experiments. When virus was detected by observation of cytopathic effects and/or hemagglutination assay, the HA CS region was sequenced by Sanger sequencing to determine the nature of the indels which resulted in frame repair and recombinant virus production. Predicted RNA stem-loops (Supplementary Fig. 1) and positive-sense orientation are used throughout the manuscript to represent the data and describe the location and nature of the indels. Ten H5_RETR_ HA_SND_s were tested and virus was detected with only three HA_SND_s in 13% (4/30) of the experiments (Fig. 1b). Two single A insertions in the A-stretch located at the 3’ end of the loop, a four-nucleotide insertion at the 3’ end and a two-nucleotide deletion in the A-stretch at the loop 5’ end were detected (Fig. 1b, Supplementary Table 1a). These insertions led to tribasic and tetrabasic CSs (RKTR, REKR and REKKR; Supplementary Table 1a).

Next, SNDs were introduced in the HA of A/Indo/5/05 (H5_MBCS_). Nineteen H5_MBCS_ HA_SND_s were tested. Virus was detected in either 2/3 or 3/3 experiments with 16 out of 19 HA_SND_s, resulting in a total of 46 detected indels. Two-nucleotide deletions (16/46), single-nucleotide insertions (15/46) and insertions of more than one nucleotide (15/46) were observed (Fig. 1c). Indels were mostly observed in the loop of the predicted H5_MBCS_ RNA structure, and only 2/46 of the indels were located at the top of the 5’ side of the stem. Indels observed in the loop were mainly located in two regions: the large A-stretch at the 3’ end of the loop (position 1057-1062) and the small A-stretch at the 5’ end of the loop (position 1041-1043). Indels that were observed repeatedly and in multiple HA_SND_s across the study were defined as indel pattern 1–4. Indel pattern 1 corresponded to a seven-nucleotide insertion (AAGAAAA) and represented 28% (13/46) of the observed indels in H5_MBCS_ HA_SND_s. Indel pattern 2 corresponded to a one A insertion in the A-stretch at the 3’ end of the loop and was observed in 26% (12/46) of indels in H5_MBCS_ HA_SND_s. Indel pattern 3 corresponded to a two-nucleotide deletion in the A-stretch at the 5’ end of the loop and was observed in 7% (3/46) of indels in H5_MBCS_ HA_SND_s. Finally, indel pattern 4 corresponded to the insertion of a single A in the A-stretch at the 5’ end of the loop and was observed in 4% (2/46) of indels in H5_MBCS_ HA_SND_s. All other detected indels are shown in Supplementary Table 1a. As many indels were observed in homopolymers, SNDs were introduced in A, U, cytosine (C) and guanine (G) homopolymers in other regions of the influenza virus genome. Virtually no indels were detected (Supplementary Table 2), suggesting a peculiarity of the CS region.

Next, SNDs were introduced in the stem of the predicted RNA structures of H5_RETR_ and H5_MBCS_. No virus was detected in any of the 33 independent experiments (Supplementary Fig. 2a, b). This indicated that either indels did not occur or remained undetected because they did not lead to frame repair or led to non-functional HA proteins.

Taken together, these results show that indels were observed at the CS of both H5_RETR_ and H5_MBCS_ albeit at a much higher level in the H5_MBCS_ HA. A striking difference between H5_RETR_ and H5_MBCS_ is the length of the A-stretch at the 3’ end of the loop, which was three and six-nucleotides long, respectively. Moreover, indels were detected in H5_RETR_ when the length of the 3’ end loop A-stretch was increased from three to four or five (HA_SND_ Δ1046 and Δ1050; Fig. 1b), suggesting that the sequence of the 3’ end of the loop might be important for indels at the HA CS.

### Increasing the length of the A-stretch at the 3’end of the loop facilitated indels in H5

**Fig. 2:**
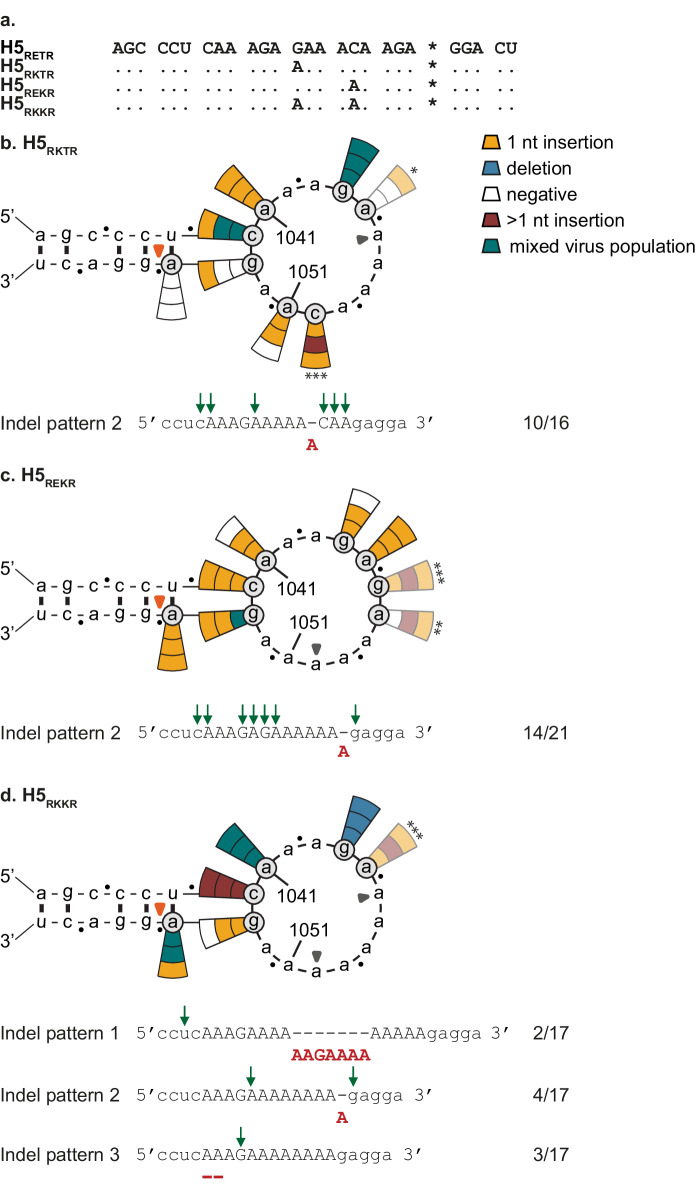
Increasing A homopolymer length facilitates indels at the H5 CS. Results are shown as described in the legend of Fig. 1. Grey closed arrow heads refer to the introduced nucleotide substitutions as compared to H5_RETR_. A green wedge indicates that the HA sequence of the rescued virus could not be reliably determined by Sanger sequencing. Faded pie slices indicate that an HA with the same sequence was previously tested and show the data from the previous experiment, identified by the same number of asterisks. Observed indel patterns and corresponding frequencies are indicated below the structures, the other detected indels are shown in Supplementary Table 1a. **a** Alignment of H5 intermediate CS sequences shown in Fig. 2. The asterisk indicates the border between HA1 and HA2. Results from testing (**b**) H5_RKTR_ HA_SND_s, (**c**) H5_REKR_ HA_SND_s and (**d**) H5_RKKR_ HA_SND_s.

Our previous observations suggested that the presence of longer A-stretches might be an important driver of indels at the HA CS (Fig. 1). To further investigate the impact of sequence on indel generation, the length of the 3’ end loop A-stretch was increased. Nucleotide substitutions were introduced in H5_RETR_ at positions 1046, 1050 or both, which increased the A-stretch length to five, six or eight nucleotides, respectively, creating tri- and tetrabasic intermediate CS sequences (G1046A (H5_RKTR_), C1050A (H5_REKR_) and G1046A/C1050A (H5_RKKR_); Fig. 2a).

Virus was detected in 67% (16/24), 88% (21/24) and 94% (17/18) of experiments using H5_RKTR,_ H5_REKR_ and H5_RKKR_ HA_SND_s, respectively (Fig. 2b–d). This contrasted with data obtained with H5_RETR_ (13%; 4/30), indicating that increasing the length of the 3’ end loop A-stretch indeed promoted indels. Most of the observed indels corresponded to the previously identified indel patterns. Indel pattern 2 was most frequently observed, being identified in 73% (10/16), 67% (14/21) and 24% (4/17) of the viruses produced using H5_RKTR_ and H5_REKR_ and H5_RKKR_ HA_SND_s, respectively. A wider variation of indels was observed when H5_RKKR_ HA_SND_s were tested (Fig. 2d and Supplementary Table 1a). Several insertions of four, seven (indel pattern 1) and even 13 nucleotides were observed, all consisting of As and one to three Gs. All insertions of 4, 7 and more nucleotides appeared to be the result of duplications of neighbouring sequences. Interestingly, a deletion of two As (indel pattern 3) was consistently observed in H5_RKKR_ HA_SND_ Δ1044, which only contained As in the loop, thereby suggesting that there may be a limit to the number of contiguous As that are tolerated at the HA CS. The HA CS sequence of some of the viruses could not be reliably determined by Sanger sequencing (indicated by a green wedge in Fig. 2), indicative of the presence of mixed virus populations. This was confirmed by performing TOPO-cloning of H5 CS PCR fragments and subsequent Sanger sequencing of six clones per fragment. A wide variety of sequences was detected (Supplementary Table 1b). In 58% (35/60) of the clones, a frameshift in the HA protein was detected due to either the presence of the introduced SND (69%; 24/35) or an indel that did not lead to reading frame repair. The most frequently observed indel was a one A insertion which resulted in a R323K amino acid change. Additionally, larger insertions of 3, 5, 7 and even 33 As were observed. Together, these data suggested that tri- and tetrabasic intermediate CS motifs were more error-prone than the LPAIV H5_RETR_ CS.

The reciprocal approach was then taken by decreasing the length of the A-stretch of the H5s for which the highest indel frequency was observed (H5_MBCS,_ H5_REKR_ and H5_RKKR_), through the introduction of G or C nucleotides leading to either silent or non-silent substitutions. In general, reducing the length of the A-stretch decreased indel frequency (Supplementary Figs. 3−6: see notes in Supplementary information). Furthermore, a higher deletion to insertion rate was detected in H5_MBCS_ with decreased A-stretch length, mostly occurring in the middle and at the 5’ end of the loop.

Taken together, these results revealed a positive association between the A-stretch length at the 3’ end of the loop and indel occurrence at the H5 CS. This suggests that the sequence of the HA CS is a key determinant of indel generation and the nature of such indels.

### Indels were observed at the H6 CS only after extensively changing the CS sequence

**Fig. 3:**
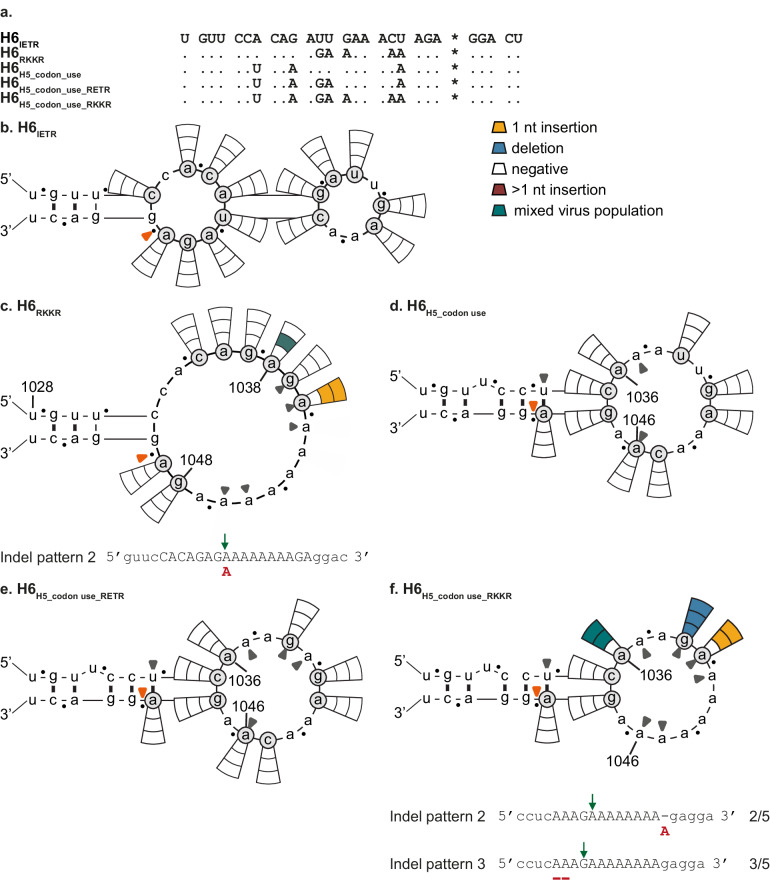
Indels are not readily detected at the H6 CS. Results are shown as described in the legend of Fig. 1. Grey closed arrow heads refer to the introduced nucleotide substitutions as compared to H6_IETR_. Observed indel patterns and corresponding frequencies are indicated below the structures, the other detected indels are indicated in Supplementary Table 1a. **a** Alignment of H6 CS sequences shown in Fig. 3. The asterisk indicates the border between HA1 and HA2. Results from testing (**b**) H6_IETR_ HA_SND_s, (**c**) H6_RKKR_ HA_SND_s, (**d**) H6_H5_codon use_ HA_SND_s, (**e**) H6_H5_codon use_RETR_ HA_SND_s and (**f**) H6_H5_codon use_RKKR_ HA_SND_s.

To gain further knowledge on subtype-restriction of MBCS acquisition, LPAIV H6_IETR_ HA_SND_s were tested in our experimental system. The HA of A/mallard/Sweden/81/2002 (H6N1; A/ml/Sw/81/02) was used as (i) no HPAIVs have so far evolved from H6 LPAIVs in nature, despite extensive circulation in poultry^37,38^ and (ii) H6 and H5 belong to the same phylogenetic group^1^ (Fig. 3a). Moreover, the H6 CS region of A/ml/Sw/81/02 corresponds to the consensus CS sequence of all H6 LPAIVs, both at the amino acid and nucleotide level^25^. SNDs were introduced into H6_IETR_ and reverse genetics experiments were performed using the H5 A/Indo/5/05 virus backbone. No virus was detected in any of the 42 experiments using H6_IETR_ HA_SND_s (Fig. 3b), reflecting the natural situation whereby no HPAIV has so far evolved from an H6 LPAIV. SNDs were also introduced into the stem of the H6 CS predicted RNA structure (Supplementary Fig. 1), and no virus was detected in any of the nine experiments (Supplementary Fig. 2c).

Next, we investigated which changes in H6_IETR_ were necessary to detect indels. Firstly, substitutions were introduced to create di- and tribasic intermediate CSs, which consequently increased the number of As at the 3’ end of the loop. To this end, the T327K substitution (C1045A and U1046A) was introduced, resulting in an IEKR CS motif and an A-stretch length of six nucleotides. Additionally, a tri-basic cleavage site, shown to be indel-prone in H5, was created by changing U1039G, U1040A, C1045A and U1046A to generate H6_REKR_ (Supplementary Fig. 7a). No indel was observed in any of the H6_IEKR_ and H6_REKR_ HA_SND_s that were tested (Supplementary Fig. 7b, c), even though a 7-nucleotide long A-stretch was present at the 3’end of the loop of H6_IEKR_ Δ1048, H6_REKR_ Δ1041 and Δ1048 HA_SND_s. This suggested that, in contrast to H5, a long A-stretch at the 3’ end of the loop might not be sufficient to facilitate indels in H6. Next, an additional G1041A substitution was introduced to create a tetrabasic CS (H6_RKKR_) (Fig. 3a), thereby increasing the length of the A-stretch to eight nucleotides. Indels were then observed in 13% (3/24) of the experiments (Fig. 3c).

**Fig. 4:**
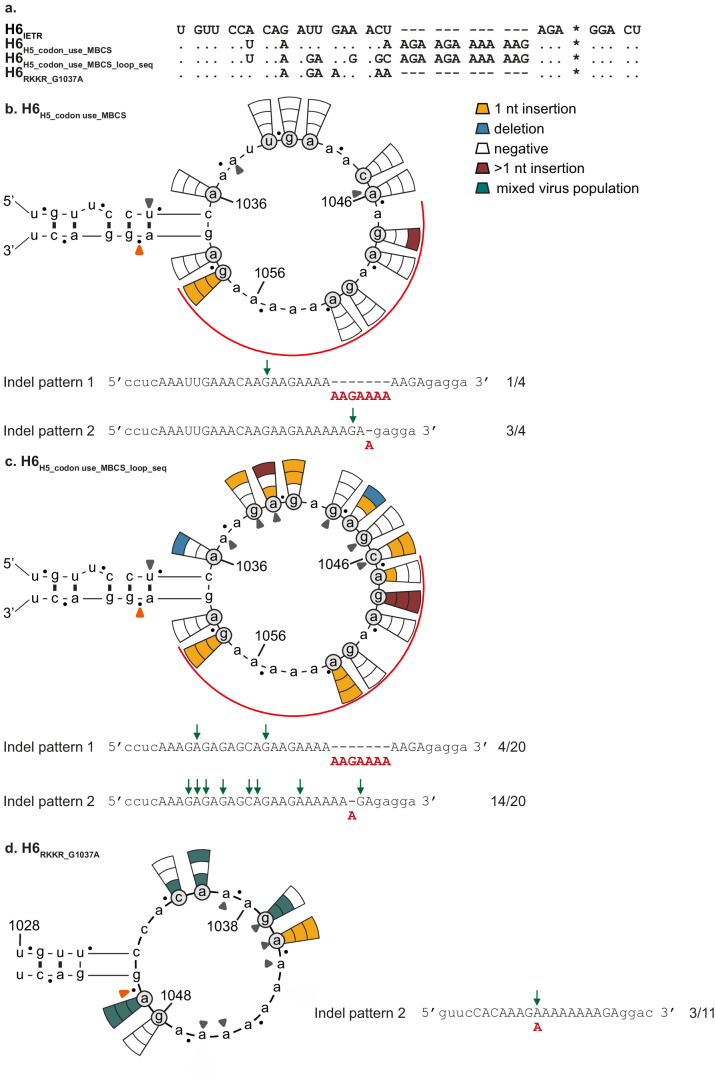
Introducing H5 loop sequences in H6 increases indel detection. Results are shown as described in the legend of Fig. 1. Grey closed arrow heads refer to the introduced nucleotide substitutions as compared to H6_IETR_. Observed indel patterns and corresponding frequencies are indicated below the structures, the other detected indels are shown in Supplementary Table 1a. **a** Alignment of all H6 HA CS sequences shown in Fig. 4. The asterisk indicates the border between HA1 and HA2. Results from testing (**b**) H6_H5_codon use_MBCS_ HA_SND_s, (**c**) H6_H5_codon use_MBCS_loop_seq_ HA_SND_s and (**d**) H6_RKKR_G1037A_ HA_SND_s. In (**b**, **c**), the red curved line indicates the location of the inserted MBCS.

As indels were either absent or occurred at a low frequency when H6 HA_SND_s were tested, we mutated the CS sequence of H6_IETR_ to resemble that of H5. In addition to a different consensus CS sequence at the amino acid level, PQRETR in H5 versus PQIETR in H6, the consensus codon use of the common amino acids differed between the two subtypes^25^. Therefore, three silent substitutions (A1034U, G1037A and U1046A) were first introduced in the A/ml/Sw/81/02 H6_IETR_ to change the P, Q and T codons to those predominantly observed in H5s from viruses of the African-Eurasian-Oceanian lineage (H6_H5_codon use_) (Fig. 3a). No indel was detected in any of the H6_H5_codon use_ HA_SND_s (Fig. 3d). Therefore, two additional non-silent substitutions (U1039G and U1040A) were introduced in H6_H5_codon use_, resulting in a loop sequence identical to that of H5_RETR_ at both the nucleotide and amino acid levels (H6_H5_codon use_RETR_). Again, no virus was detected in any of the experiments using H6_H5_codon use_RETR_ HA_SND_s (Fig. 3e). Next, additional non-silent substitutions (G1041A and C1045A) were introduced to change the sequence of the H6_H5_codon use_RETR_ loop to that of H5_RKKR_ (H6_H5_codon use_RKKR_). Virus was detected in 39% (7/18) of experiments (Fig. 3f), a higher frequency than that observed for H6_RKKR_ (13%). The indels that repaired the HA reading frame corresponded to pattern 2 and 3, which were also observed multiple times in H5. The CS sequence of the virus obtained using H6_H5_codon use_RKKR_ HA_SND_ Δ1036 could not be reliably detected by Sanger sequencing and sequencing of a subset of TOPO clones revealed a mixed virus population, as observed for H5_RKKR_ (Supplementary Table 1b). As a high indel frequency was observed in H5_MBCS_, the nucleotides that encode the A/Indo/5/05 H5 MBCS (AGA AGA AAA AAG; RRKK) were introduced into H6_H5_codon use_ between T327-R328 (H6_H5_codon use_MBCS_) (Fig. 4a). Virus was detected in only 11% (4/36) of the experiments (Fig. 4b). Additional substitutions (U1039G, U1040A, A1043G, C1045G and A1046C) were introduced in H6_H5_codon use_MBCS_ to change the loop sequence completely to that of H5_MBCS_ (H6_H5_codon use_MBCS_loop_seq_) (Fig. 4a). This increased virus detection frequencies from 11 to 44% (20/45; Fig. 4c). Most of the indels observed in H6_H5_codon use_MBCS_ and H6_H5_codon use_MBCS_loop_seq_ HA_SND_s corresponded to the previously identified patterns 1 and 2, which were also observed frequently in H5_MBCS_.

Upon increasing the length of the A-stretch at the 3’ end of the loop and inserting an H5 MBCS into the H6 CS, indel frequencies remained low. In addition, indel frequency in H6 HA_SND_s containing the RKKR CS was higher in the presence of the H5 codon use substitutions (13 versus 39%). These HAs only differed by two substitutions, and we sought to investigate the impact of the substitution closest to the CS (G1037A) (Fig. 4a). Introducing G1037A into H6_RKKR_ (H6_RKKR_G1037A_) HA_SND_s increased indel frequency from 13 to 61% (11/18; Fig. 4d), which was also higher than observed in the H6_H5_codon use_RKKR_ HA_SND_s (39%). Of note, 1037A, although not present in the H6 LPAIV CS consensus, is found in 37.4% of H6 viruses^25^. Indels identified by Sanger sequencing in H6_RKKR_G1073A_ HA_SND_s corresponded to indel pattern 2. Nevertheless, as previously observed for H5_RKKR_, H6_RKKR_ and H6_H5_codon use_RKKR_, the CS sequence of most viruses could not be reliably determined by Sanger sequencing, and a mixed virus population was revealed upon sequencing of TOPO-clones (Supplementary Table 1b). The majority (75%; 33/44) of the sequences showed a frameshift in HA, partially due to the presence of the introduced SND (39%; 13/33). The in-frame insertions mostly led to an additional K in the CS, and a ten A insertion, creating a PQRKKKKKKG CS, was observed.

### No direct relationship between indel detection rate and particle production efficiency was observed

In order to investigate whether differences observed in indel frequency could be due to differences in particle production upon reverse genetics, plaque forming units per ml (PFU/ml) were measured. Madin-Darby Canine Kidney (MDCK) cells were inoculated with 293 T supernatants derived from reverse genetics experiments with all HA plasmids used as templates to produce HA_SND_s, to the exception of those coding for HAs with silent substitutions. Virus titres of H5_MBCS_ or H5_RKKR_ viruses were higher than those of viruses with dibasic (H5_RETR_) and tribasic (H5_REKR_ and H5_RKTR_) CSs, probably due to cleavage independent of trypsin allowing re-amplification in 293T cells (Supplementary Fig. 8). Therefore, the increased indel detection in H5_MBCS_ and H5_RKKR_ HA_SND_s could be partially due to a higher rescue efficiency and the opportunity for additional rounds of replication. On the other hand, virus titres of H5_REKR_ and H5_RKTR_ viruses were comparable to that of the H5_RETR_ virus even though indels were detected more frequently in H5_REKR_ and H5_RKTR_ than in H5_RETR_ HA_SND_s.

In general, virus titres of H6 viruses were lower than those of H5 viruses. The number of particles produced upon rescue of H6_H5_codon use_MBCS_ and H6_H5_codon use_MBCS_loop_seq_ viruses was slightly lower than for the H5_MBCS_ virus, perhaps due to lower cleavage efficiency resulting in lower re-amplification levels. Nevertheless, they were higher than those of the H5_REKR_ and H5_RKTR_ viruses, for which high indel frequencies were observed. Virus titres of H6_RKKR_, H6_H5_codon use_RKKR_ and H6_RKKR_G1037A_ viruses were similar or lower than that of the H6_IETR_ virus, yet increased indel detection was observed in the formers. The lowest virus titre was observed for the H6_REKR_ virus, and thus it cannot be excluded that the absence of indels in H6_REKR_ was partially the result of low virus rescue efficiency. Taken together, these results show that no direct relationship between indel detection rate and virus titre was observed. However, it cannot be fully excluded that, for some HAs, indels were not observed because of low virus production and insufficient rounds of genome replication by the RdRp.

### *Trans*-complementation with a functional H5 protein increased indel detection in the H5 CS but not in that of H6

As a low number of indels were detected using H5_RETR_ HA_SND_s and no indel was detected with many H6 HA_SND_s, a *trans*-complementation experiment was performed to attempt to increase indel detection sensitivity. To this end, an H5_RETR_ expression plasmid was co-transfected in 293T cells along with the eight reverse genetics plasmids. This will result in the expression of a functional HA protein, to ensure that infectious virus particles harbouring HA_SND_ genes are produced in 293T cells. An additional round of replication in MDCK cells can then take place, thus increasing the opportunity for indel generation by the RdRp. Addition of the H5_RETR_ expression plasmid during transfection of the H5_RETR_ HA_SND_s led to an increase in the number of indels that were detected (Fig. 1b and Supplementary Fig. 9a). *Trans*-complementation decreased the clonality of the rescued viruses, whose CS sequences could not be accurately determined using Sanger sequencing. Therefore, six TOPO-clones of HA PCR fragments amplified from MDCK supernatants with an HA titre of ≥8 hemagglutination units (HAU/25 µl) were sequenced. An HA-titre of 8 HAU/25 µl was chosen as it was the lowest titre which coincided with the observation of cytopathic effect in MDCK cells, suggesting virus replication rather than just efficient *trans*-complementation of deficient viruses with an HA_SND_. TOPO-clones contained different sequences, indeed indicative of the presence of mixed virus populations (Supplementary Table 1b). In total, a positive HA titre of >8 HAU/25 µl was observed with 19/30 (63%) HA_SND_s, while this was only 13% (4/30) without *trans*-complementation. Frameshift was observed in 11% (13/119) of the clones, which was mostly due to the presence of the introduced SND. In some occasions, the WT sequence was detected, which could be due to H5_RETR_ expression plasmid DNA detection, despite extensive DNAse treatment and negative -RT controls. In total, for seven samples, either only the SND, only the WT, or a combination of both WT and SND were detected in all 6 clones. For one sample, TOPO-cloning failed and thus no sequence was obtained. Other one-nucleotide insertions and deletions were also detected. Taken together, indel detection of H5_RETR_ HA_SND_s increased upon *trans*-complementation. A similar approach was taken using all H6s in which no indel had been detected (H6_IETR,_ H6_IEKR_, H6_REKR_, H6_H5_codon use_ and H6_H5_codon use_RETR_ HA_SND_s). An HA-titre of ≥8 HAU/25 µl was detected only in 5% (2/42) and 8% (1/12) of experiments with H6_IETR_ and H6_H5_codon use_RETR_ respectively (Supplementary Fig. 9b−f) and only the introduced SND was observed upon Sanger sequencing of the corresponding clones, probably due to very efficient *trans*-complementation. Taken together, these data show that *trans*-complementation increased indel detection in H5, but not in H6.

## Discussion

To date, the exact molecular mechanisms underlying MBCS acquisition and the reason(s) why HPAIVs have only evolved from LPAIVs of the H5 and H7 subtypes remain unknown. As reproducing MBCS acquisition in viruses in a laboratory setting has been proven very difficult, we developed a novel sensitive, virus-based system to study the drivers of insertions at the HA CS. Using this system, indels were detected relatively frequently. We observed that A-stretches at both the 5’ and 3’ end of the loop were the main regions where indels occurred. In addition, four indel patterns were observed, present in 54% (121/226) of the indel-containing viruses. At the 3’ end of the loop, 77% (98/127) of indels were insertions, suggesting that insertions in this part of the loop are well tolerated at the RNA and protein levels. At the 5’ end of the loop, mostly one-nucleotide insertions (11/37) or two-nucleotide deletions (12/37) were observed, suggesting that larger insertions at this location might be detrimental at the RNA or protein level. Accordingly, reducing the length of the A-stretches in the H5_MBCS_ loop resulted in an increased deletion to insertion ratio that occurred at the 5’ end of the loop. Of note, most of the insertions of more than one nucleotide contained one G (32/36) among six (24/36) or three (8/36) As. A-stretches interrupted by Gs might be more prone to duplication through backtracking and re-alignment than A-stretches interrupted by pyrimidines, as Us in the product can pair with both As or Gs in the template during realigning, as previously suggested by Perdue et al.^18^. A six-nucleotide direct repeat consisting of five As and one G has also been observed in previous studies, which either occurred in nature or after in ovo passaging^11,18,39^.

Data from the present study show that nucleotide sequence is an important driver of indels. We observed that increasing the A content in H5_RETR_, even through only one nucleotide substitution, increased indel frequency. Of note, it remains unknown whether MBCSs are generated during cRNA and/or vRNA replication and therefore if A and/or U content is a driver of insertions. Our data are in accordance with that of previous studies^20,22,24,26,34,36^. It was observed in ovo, in vivo and in vitro that H5 viruses with an intermediate CS genotype and higher A content, i.e., REKR, RKTR or RKKR, acquired insertions at the CS more easily than viruses with the consensus RETR CS^22,24,26,34,36^. The importance of polypurine-rich regions in insertion acquisition at the H5 CS region has also been proposed by Perdue et al.^18^. Using our experimental system, a trend towards fewer indels was observed upon reducing the length of the A-stretch, even when interrupting it with Gs, suggesting that nucleotide nature (A,C,G,U) rather than base type (purine/pyrimidine) might be a determinant.

This study revealed novel insights regarding the subtype-restriction of MBCS acquisition. Indel detection in H5 increased upon a single nucleotide substitution, when the H5 CS was changed from RETR to REKR or RKTR. In contrast, indels in H6 were only observed when five or more nucleotide substitutions were introduced in the H6 CS. Of note, our experimental system is not fully quantitative, and it cannot be completely excluded that differences in rescue efficiency might lead to small variations in indel detection sensitivity between H5 and H6. Data from the present study are in line with observations from an in-silico analysis that we recently conducted using a comprehensive dataset of all available LPAIV HA CS sequences from all 16 HA subtypes^25^. It was observed that fewer substitutions were required in H5 and H7 HA CS sequences to obtain an insertion-prone sequence, as defined by the presence of three basic amino acids or A-stretches, than in those from other subtypes, including H6. Collectively, these results point to the fact that several substitutions are necessary to generate indels in H6 as a potential limitation for MBCS acquisition in non-H5/H7 subtypes. In addition, while nucleotide sequences of intermediate CS used in this study, e.g., H5_REKR_ and H5_RKTR,_ have been detected in WT virus isolates at low frequencies, none of the H6 sequences in which indels were detected in the present study have been observed in natural isolates^25^. Some non-H5/H7 viruses with tribasic CSs have been isolated (e.g., H4N2 and H9N2)^23,40–43^, yet they remained trypsin-dependent in vitro^40,41^. The tribasic CS in an H4 virus was acquired by a substitution and insertion, and the tribasic CSs in H9 viruses were acquired by substitutions only. Previously, Zhang et al. showed that changing the H9N2 HA CS to a tri- or tetrabasic CS, thereby increasing the number of consecutive As, increased the detection of insertions as indirectly measured by luciferase activity in a minigenome assay^23^, corroborating that the nucleotide sequence of the CS is a key determinant of insertions. Nevertheless, indel detection in H6 was never as high as in H5 in our study, even when the full H5_RKKR_ or H5_MBCS_ sequence was introduced in H6. Although it cannot be fully ruled out that this could be partially due to a lower particle production in H6 compared to H5, this observation suggests that RNA regions outside of the CS and other factors than CS nucleotide sequence, e.g., RNA structures, might play a role in MBCS acquisition. Several conserved alternative configurations of putative RNA stem-loop structures that encompass CS codons in H5 and H7 HA sequences have been predicted using folding algorithms on naked protein-free RNA, supported by covariation and phylogenetic analyses^19,21,44^. Although it has been proposed that RNA folding in the CS region might drive MBCS acquisition^11,18,22–24,45^, strong empirical evidence is lacking. Reducing the loop size of these predicted RNA CS structures, yet retaining a large A-stretch of 12 or nine nucleotides in H5 or H9 HA CSs respectively, reduced indels as indirectly measured by a luciferase-based minigenome assay^22,23^. Kida et al. reported a reduced indel frequency when the length of the A-stretch and the size of the predicted loop were decreased simultaneously, precluding the discrimination between the impact of these two factors on indels^24^. Here, we refrained from investigating the role on MBCS acquisition of these putative RNA structures in MBCS acquisition. These structures are predicted in-silico on naked RNA and might reflect those present in quiescent vRNPs. Instead, it might be more relevant to analyse structures in the context of RNA replication by the influenza RdRp, as RNA structures are expected to be melted by the polymerase complex during replication and might also be altered in the presence of the nucleoprotein.

One limitation of the presently used system is that only frame-restoring indels (insertion of 3n + 1 or deletion of 3n −1 nucleotides) resulting in a functional HA protein are detected. Consequently, the lack of detection of replication competent viruses does not necessarily mean that indels did not occur at all. Other indels not leading to reading frame repair or leading to sequences detrimental to HA structure, folding, expression or function might have occurred but would not be detected using this experimental approach. The development of very sensitive, controlled and reliable next-generation sequencing methods and analyses that allow for the accurate detection of all indels, especially in homopolymer regions, is crucial. Unfortunately, the most widely used next-generation sequencing methods are still unreliable when it comes to the detection of indels in homopolymer regions, which are error-prone for many replication enzymes^46^. Another potential limitation is that our experimental system, based on reverse genetics, relies on the human RNA polymerase I (Pol I) for initial transcription of viral genomic RNA from the transfected plasmids^47^. Therefore, it cannot be fully excluded that some of the observed indels might have occurred during plasmid transcription. The insertion and deletion rates of Pol I from yeast, estimated at 8.8 × 10^−7^ and 3.4 × 10^−7^ per base pair respectively, have been recently investigated using circular sequencing^48^. It was observed that the majority of insertions were one or two nucleotides in length and that, as expected, homonucleotide and dinucleotide tracts were hotspots for indels by the yeast RNA polymerase II, as it is the case for many replication enzymes. Nevertheless, it seems unlikely that Pol I errors majorly contribute to the present data as: (i) indels differed in pattern and length including multiple insertions of four or more nucleotides (30%; 40/133), (ii) no indel was detected in many HA_SND_s, despite the presence of long homopolymers (notably in H6 HAs and in the homopolymer controls) and (iii) the addition of an MBCS, and thus an A-stretch, in H6 only marginally increased indels, while changing the 5’ end of the loop of H6_H5_codon_use_MBCS_loop_seq_ to that of H5, without the addition of homopolymers, increased indels.

We have here developed a novel and sensitive experimental setup to study drivers of MBCS acquisition and showed that nucleotide sequence is a key determinant driving insertions at the H5 CS. We have shown for the first time that insertions can be detected at the H6 CS when substitutions increasing the A/U content were introduced. This experimental approach could be further used to identify drivers of indels at the CS of HAs of viruses from other subtypes than H5 and H6. Moreover, the impact of nucleotide substitutions within the CS region could be investigated to identify the minimal number of nucleotide substitutions needed for non-H5 and -H7 viruses to acquire a MBCS. This information could then be used in surveillance programmes to flag LPAIV with potential to evolve to HPAIV.

## Methods

### Cells

MDCK cells were cultured at 37 °C and 5% CO_2_ in EMEM (Capricorn) supplemented with 1.5 mg/ml sodium bicarbonate (Gibco), 10 mM HEPES (Capricorn), 100 IU/ml penicillin (Capricorn), 100 µg/µl streptomycin (Capricorn), 2 mM glutamine (Capricorn), 1X non-essential amino acids (Capricorn) and 10% fetal calf serum (FCS). Human epithelial 293T cells were cultured at 37 °C and 5% CO_2_ in DMEM (Capricorn) and supplemented with 1 mM sodium pyruvate (Gibco), 100 Iµ/ml penicillin, 100 µg/µl streptomycin, 2 mM glutamine, 1X non-essential amino acids and 10% FCS. During basal cell culture 293T cells were maintained in the presence of 500 µg/ml geneticin (Gibco) and passaged when sub confluent.

### Plasmids

The generation of reverse genetics plasmids (modified version of pHW2000) containing all gene segments of A/Indo/5/05 and the HA of A/ml/Sw/81/02 (accession number: MN515099.1) was described previously^49,50^. The A/Indo/5/05 virus was kindly provided by M. Peiris (Hongkong University). The MBCS of the A/Indo/5/05 HA was removed (H5_RETR_) as described^50^. The generation of reverse genetics plasmids coding for the HA and NA segments of A/PR/8/1934 (H1N1), the HA and M segments of A/Netherlands/602/2009 (H1N1), the HA segment of A/WSN/1933 (H1N1) and the HA segment of A/Guangzhou/39715/2014 (H5N6) were described previously^47,51–53^. The HA gene segment of the H5 A/Indo/5/05 ΔMBCS (H5_RETR_) was cloned into the pCAGGS expression vector, which was kindly provided by Dr. A. Garcia-Sastre (Icahn School of Medicine, New York, U.S.A.).

The plasmids containing the H5_RETR_, H5 A/Indo/5/05 (H5_MBCS_), and H6 A/ml/Sw/81/02 (H6_IETR_) HA were used as templates for site-directed mutagenesis using PFU Ultra II (Agilent Technologies) as described previously^54^. All HA plasmids were confirmed to lead to the expression of functional HA proteins and viruses when used in reverse genetics experiments and were subsequently used as templates to introduce SNDs. Every nucleotide belonging to the H5 LPAIV CS (Q322-R326), H5 HPAIV CS (Q322-R330), H6 CS (Q340-R344) or H6_H5_codon_use_MBCS_ CS (nucleotide 1036-1059) resulting in a unique sequence, were deleted in the HAs described in the main text. A subset of SNDs were introduced into the H5 HAs containing reduced A-stretch lengths. All introduced nucleotide substitutions are indicated in the figures by grey arrowheads and sequences of all the CSs used to introduce SNDs can be viewed in Supplementary Table 1a, c. The presence of the desired mutation in each plasmid preparation was confirmed by Sanger sequencing using a 3130XL or 3500XL genetic analyser (Applied Biosystems). Respective H5 or H6 numberings, starting from the signal peptide, are used throughout the manuscript. Primer sequences are available upon request.

### Recombinant virus production and sequencing

Recombinant viruses were produced essentially as described previously^51^. In brief, 293T cells were transfected with 5 µg of a reverse genetics plasmid coding for WT HA or HA_SND_s, together with 5 µg each of the remaining H5N1 A/Indo/5/05 reverse genetics plasmids (H5 virus backbone). Three days after transfection, undiluted 293T supernatant was used to inoculate MDCK cells and virus production was assessed after three days by hemagglutination assay with 1% turkey red blood cells. If virus was detected, RNA was extracted from 200 µl of the supernatant with the high pure RNA isolation kit (Roche), according to the instructions of the manufacturer. Complementary DNA (cDNA) was produced by reverse transcription (RT) using Superscript IV (Invitrogen) and an influenza A virus specific primer (5’-*AGCRAAAGCAGG*) according to the instructions of the manufacturer. The region in HA spanning the HA CS was amplified by CS PCR using PFU Ultra II and primers 5’-*GGCGATAAACTCTAGTATGC-3’* and 5’-*CGGATAGTTGTACGTTCCGT-3’* for H5, and 5’-*GGTAACAAAAGCTTGCCCTT-3’* and 5’-*ATTCGTGGTCGACAGCTTCG-3’* for H6. PCR products were visualised by gel electrophoresis and agarose bands containing DNA were extracted and purified using the MinElute gel extraction kit (Qiagen) according to the instructions of the manufacturer. Next, sequences of PCR products were obtained by Sanger sequencing using a 3130XL or 3500XL genetic analyser. If ambiguous sequences were obtained by Sanger sequencing, thereby suggesting a mixed virus population, the purified HA band resulting from the H5- or H6-specific CS PCR was cloned using the Zero Blunt TOPO PCR cloning kit (Invitrogen) according to the instructions of the manufacturer. For each sample, 6 clones were sequenced using Sanger sequencing. Samples were considered negative when the Sanger sequencing of all TOPO-clones revealed the presence of the introduced SND, except for the *trans*-complementation samples.

### *Trans*-complementation experiments

*Trans*-complementation assays were performed as described above with the additional co-transfection of 5 µg of a pCAGGS expression plasmid coding for the H5_RETR_ along with the eight reverse genetics plasmids. In order to deplete plasmid DNA, the MDCK supernatant was passed through a 0.45 µm filter to remove cell debris and the isolated RNA was subjected to extensive DNAse treatment. First, the DNAse I treatment from the high pure RNA isolation kit (Roche) was extended to 30 min. Subsequently, 1 µl of DpnI was added to the extracted RNA followed by incubation for 1 h at 37 °C and DpnI inactivation for 20 min at 80 °C. Turbo DNAse treatment and bead inactivation (Invitrogen) were subsequently performed according to the instructions of the manufacturer. Next, cDNA and HA amplicons were produced as described above. A minus RT control, in which 3 µl of RNA was directly added to the PCR mix, was included in the subsequent HA PCR to confirm the complete removal of plasmid DNA.

### Plaque assays

Plaque assays were performed to determine the particle production efficiency upon reverse genetics in 293T cells with most H5 and H6 HAs that were used as templates for the introduction of SNDs. For the mutants containing reduced A-stretch lengths, only the constructs containing non-silent substitutions were tested. The plaque assay was essentially performed as described previously^54^. In brief, different dilutions of 293T supernatant were added to 6-well plates with confluent MDCK cells. After one hour of incubation at 37 °C and 5% CO_2_, the inoculum was removed and cells were washed with PBS twice. Subsequently, 4 ml of a 1:1 dilution of Avicel RC-591 (IMCD) in 2x EMEM (Capricorn) with N-tosyl-L-phenylalanine chloromethyl ketone treated trypsin (Sigma) was added to the plates. Twenty-eight hours after inoculation, the cells were washed twice with PBS and subsequently fixed with 80% acetone. NP staining was performed as described previously^54^ and the number of plaques were counted using ImageQuant TL colony counting software version 8.2.0.0 (GE Healthcare, Life sciences).

### Biosafety

All experiments with H5N1 and H6N1 viruses were performed under biosafety level 3 or 3^+^ conditions.

### RNA structure predictions

The predicted conserved RNA structures in the H5 and H6 CS region were described previously^19,21^. RNA structures of the modified H5 and H6 CS stem-loop region and of HA_SND_s were predicted using the UNAFold Web Server (http://www.unafold.org/mfold/applications/rna-folding-form.php) with default settings. All RNA structure predictions are shown in the positive sense orientation and the indicated 5’ and 3’ ends of the loop are based on the positive sense orientation.

## Supplementary information

Supplementary Information

Supplementary Table 1

## Data Availability

All relevant data are provided within the manuscript and its Supporting Information files. Additional data are available from the corresponding author (M.R.) on request.
