# Supplementary material for: Nucleotide sequence as key determinant driving insertions at influenza A virus hemagglutinin cleavage sites
Source: Npj Viruses. 2024 May 13;2:17. doi: 10.1038/s44298-024-00029-1 (PMC11721075; doi:10.1038/s44298-024-00029-1)
Supplement: Supplementary file 1 — Supplementary Information [file 44298_2024_29_MOESM1_ESM.pdf]

Supplementary information

Index

Supplementary legend Table 1	2
Supplementary Table 2	3
Supplementary Figure 1	4
Supplementary Figure 2	5
Supplementary notes supporting Supplementary figures 3-7	6
Supplementary Figure 3	7
Supplementary Figure 4	8
Supplementary Figure 5	9
Supplementary Figure 6	10
Supplementary Figure 7	11
Supplementary Figure 8	11
Supplementary Figure 9	12
References	13

Legend supplementary Table 1

This table shows an overview of all HA_{SND}s tested. Supplementary Table 1a shows the data described in the main text, Supplementary Table 1b shows the results of TOPO-cloning and Supplementary Table 1c shows data described in the supplementary information. In column **A**, the name of the HA template is shown and in column **B** the identifier of the corresponding sequence is indicted. In column **C**, the HA CS sequence of the WT HA (in grey), the template containing the SND and of the produced virus is shown for each independent reverse genetics experiment or TOPO-clone. The location of the SND is indicated by an "X". As the exact location of the indel, in most cases, could not be unambiguously allocated, insertions were aligned at the far-right and deletions at the far-left part of the sequence. If a one nucleotide insertion was located within a homopolymer, the insertion was positioned at the location of the SND (X). Absence of detection of viable recombinant virus is indicated by "NEG"; no data indicates that the sequences obtained by Sanger sequencing were unreliable and subsequent TOPO-cloning was performed. ND= not done and NA= not applicable. In column **D,** the resulting CS motif or the WT CS motif without the SND are indicated with grey lines. Amino acids alignments were performed independently from the nucleotide alignments by running ClustalW in Bioedit (version 7.2.5) using default settings. The asterisk in the CS motif indicates the site of cleavage between HA1 and HA2. Column **E** shows the indel type (ins: insertion; del: deletion; SND: obtained sequences is identical to that of the HA_{SND} template that was used; NA= not applicable). Column **F** indicates the indel pattern which the observed indel corresponds to.

Supplementary Table 2. Results obtained from reverse genetics experiments using gene segments with SND in A, C, G or U homopolymers. The sequence flanking the homopolymer is indicated and the homopolymer is shown in bold. Green arrows indicate the nucleotide which was deleted. The acquired indel is indicated in red. The results of three independent reverse genetics experiments are shown.

**Sequence**	**Gene (nucleotide position*)**	**Replicates**		
		**1**	**2**	**3**
gugaac**aaaaaa**gggaaa	HA[a] (582-587)	NEG	NEG	**aaaaag**ggg
gucuguc**aaaaa**ugguac	HA[b] (1506-1510)	NEG	NEG	NEG
aucguc**uuuuuuu**caaau	M[c] (850-856)	NEG	NEG	NEG
aggacc**uuuuuu**cugacc	NA[a] (369-374)	NEG	NEG	NEG
caugaa**gggggg**uggaca	HA[c] (1098-1103)	NEG	NEG	NEG
uuuuugacuca**ggggg**cu	NA[b] (428-432)	NEG	NEG	NEG
ugucaaaca**cccc**aggga	HA[d] (924-927)	NEG	NEG	NEG
augacua**ccccc**aguauu	HA[e] (1540-1544)	NEG	NEG	NEG

[a] from A/PR/8/1934 (H1N1), [b] from A/mallard/Sweden/81/2002 (H6N1), [c] from A/Netherlands/602/2009 (H1N1), [d] from A/WSN/1933 (H1N1) and [e] from A/Guangzhou/39715/2014 (H5N6). * = counted from the first nucleotide of the non-coding region, NEG = negative

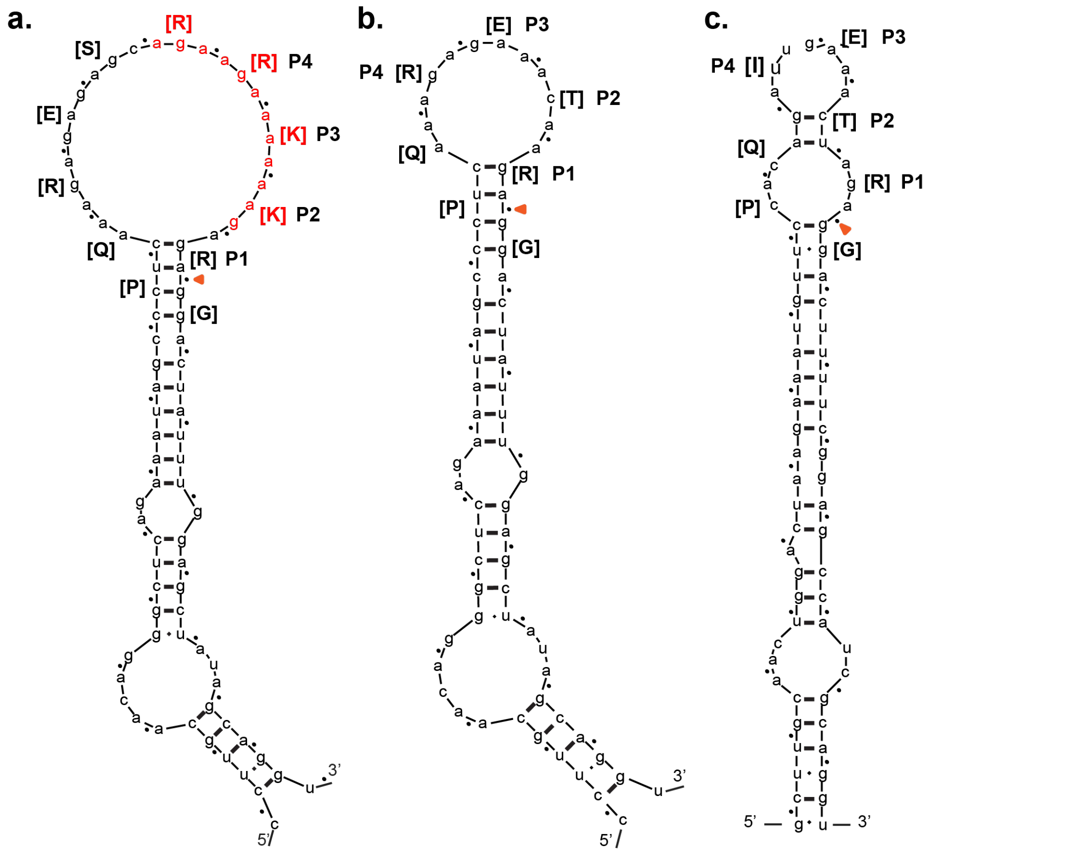

$H5_{MBCS}$
A/Indonesia/5/2005(H5N1)

$H5_{RETR}$
A/Indonesia/5/2005(H5N1)

$H6_{IETR}$
A/mallard/Sweden/81/2002(H6N1)

Supplementary Figure 1. Predicted cRNA secondary RNA structures at the H5 and H6 CS. Minimum free energy secondary RNA structures of the cRNA regions containing the HA CS region were predicted using the mfold algorithm with default settings [1, 2]. All RNA structures were predicted in a positive sense RNA orientation. The black dots delineate codons. The orange arrow head indicates the start of the N-terminal HA2 glycine amino acid residue codon. P1, P2, P3 and P4 represent the amino acid residues next to the N-terminal HA2 glycine amino acid residue, with P1 being the residue adjacent to the cleavage site. (a) Wild-type H5N1 A/Indonesia/5/2005 HA ($H5_{MBCS}$). The sequence in red represents nucleotides that encode additional basic amino acids in the MBCS. The predicted stem-loop structure spans nucleotides 1012-1090; free energy (ΔG): -22.40 kcal/mol. (b) An LPAIV HA that contains the H5 amino acid ($H5_{RETR}$) and nucleotide consensus CS motif was generated by knocking out the MBCS from $H5_{MBCS}$. The predicted stem-loop structure spans nucleotides 1012-1078; ΔG: -23.10 kcal/mol. (c) H6N1 A/mallard/Sweden/81/02 HA ($H6_{IETR}$). The predicted stem-loop structure spans nucleotides 1007-1073; ΔG: -17.50 kcal/mol.

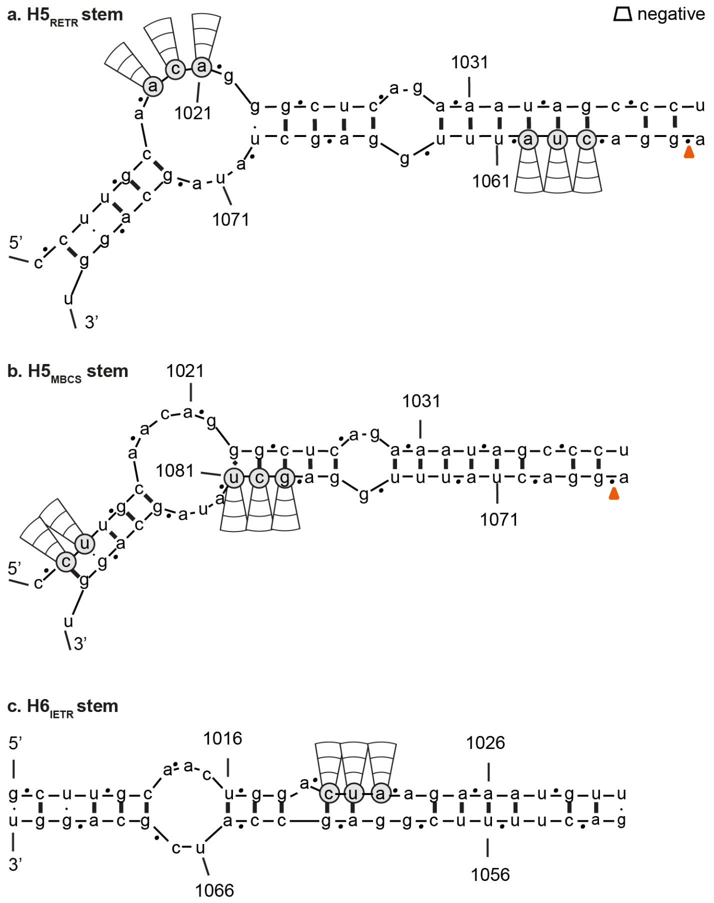

Supplementary Figure 2. No indels were observed upon reverse genetics experiments using HAs with single nucleotide deletion in the H5$_{RETR}$, H5$_{MBCS}$ and H6$_{IETR}$ stems. Results are shown as described in the legend of Figure 1. (a) Results from testing H5$_{RETR}$ HA$_{SND}$s, (b) H5$_{MBCS}$ HA$_{SND}$s and (c) H6$_{IETR}$ HA$_{SND}$s.

Supplementary notes supporting supplementary figures 3-6

Decreasing the length of the A-stretch at the 3' end of the loop reduced indel detection.

The six-nucleotide A-stretch at the 3' end of the loop of $H5_{MBCS}$ HA was interrupted by the introduction of the A1060G substitution ($H5_{MBCS\_A1060G}$), leading to a maximum of three consecutive As, or by changing the nucleotide composition of codon R327 from AGA to CGU ($H5_{MBCS\_A1055C\_A1057U\_A1060G}$), a codon that is never observed in basic amino acids of HPAIVs CSs (data not shown). A trend towards an increased deletion to insertion ratio was observed (Supplementary Fig. 3) compared to $H5_{MBCS}$ (Fig. 1b). Next, the A-stretch at the 3' end of the loop was interrupted by introducing non-silent A1059C (K328T) and/or A1062C (K329T) substitutions in $H5_{MBCS}$. The introduced substitutions changed the CS motif to RESRRTKR, RESRRKTR or RESRRTTR, respectively. Of note, H5 HPAIVs with a T at position P2 (Supplementary Fig. 1) within the CS have been detected in nature, with RKRKTR or RKRKRKTR CS motifs [3, 4]. Compared to $H5_{MBCS}$, indel detection was reduced in $H5_{MBCS\_A1059C}$ and $H5_{MBCS\_A1059C\_A1062C}$ HA_{SND}s, but less so in $H5_{MBCS\_A1062C}$ HA_{SND}s. This was especially striking for the $H5_{MBCS\_A1059C\_A1062C}$ HA_{SND}s (Fig. 1b and Supplementary Fig. 4), as indel frequency dropped to 22% (4/18) compared to 100% (18/18) observed in $H5_{MBCS}$.

To investigate the impact of the length of the A-stretch at the 3' end of the loop on indels in $H5_{REKR}$ and $H5_{RKKR}$ HAs, it was reduced by introducing silent A to G substitutions. A lower indel frequency was observed in $H5_{REKR}$ (Supplementary Fig. 5). However, little to no impact on indel detection was observed in the mutated $H5_{RKKR}$ HA_{SND}s (Supplementary Fig. 6).

Additionally, plaque assays with 293T supernatant from reverse genetics experiments with HAs carrying non-silent substitutions ($H5_{MBCS\_A1062C}$, $H5_{MBCS\_A1059C}$ and $H5_{MBCS\_A1059C\_A1062C}$) were performed. Viral particle production was not affected (Supplementary Fig. 8), suggesting that the reduced indel frequency was not caused by a lower virus rescue efficiency.

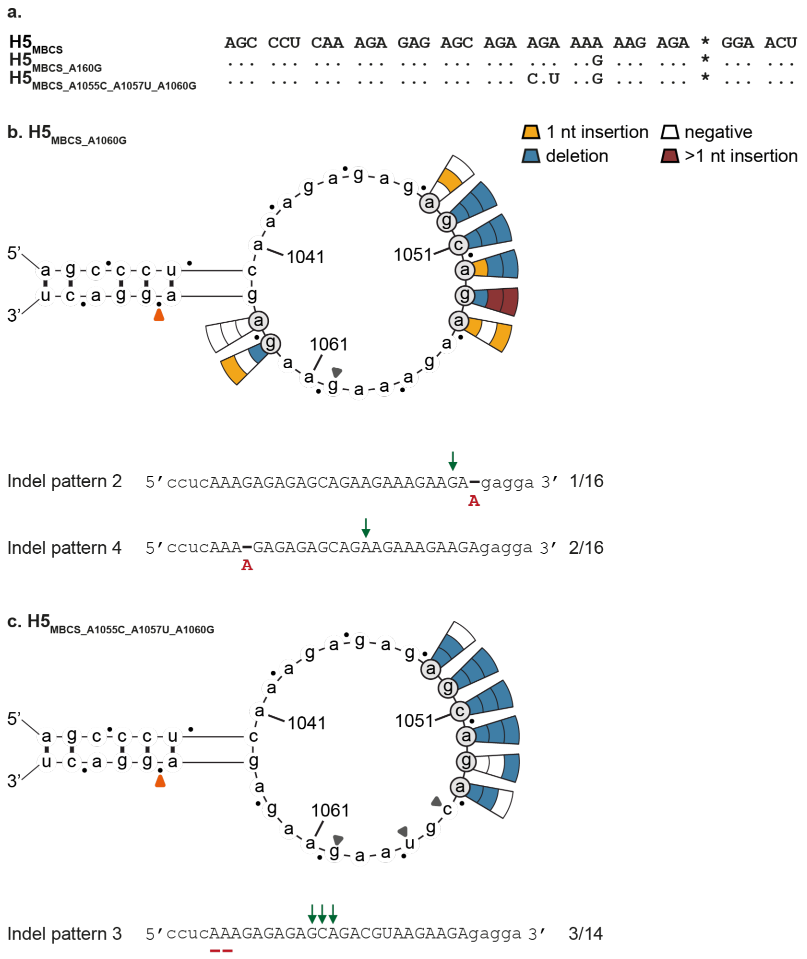

Supplementary Figure 3. Increased deletion to insertion ratio was observed upon decreasing the A-stretch length at the 3' end of the H5MBCS loop by introducing silent substitutions. Results are shown as described in the legend of Figure 1. Grey closed arrow heads refer to the introduced nucleotide substitutions as compared to H5MBCS. Observed indel patterns and corresponding frequencies are indicated below the structures, the other detected indels are shown in Supplementary Table 1c. (a) Alignment of H5MBCS CS sequences shown in Supplementary Figure 3. Results from testing (b) H5MBCS_A1060G HASNDs and (c) H5MBCS__A1055C_A1057U_A1060G HASNDs.

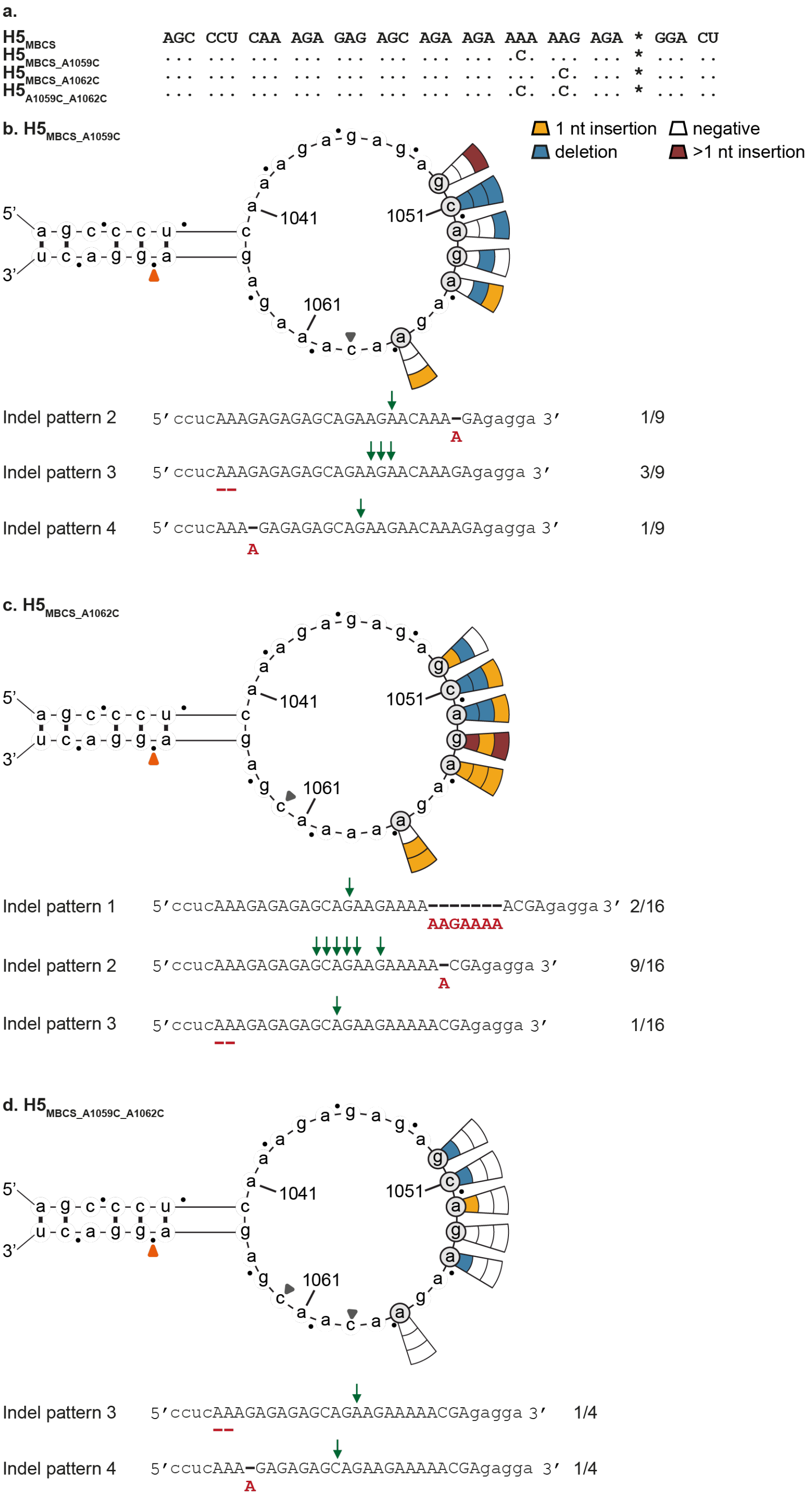

Supplementary Figure 4. Fewer indels were observed upon decreasing the A-stretch length at the 3' end of the H5MBCS loop by introducing non-silent substitutions. Results are shown as described in the legend of Figure 1. Grey closed arrow heads refer to the introduced nucleotide substitutions as compared to H5MBCS. Observed indel patterns and corresponding frequencies are indicated below the structures, the other detected indels are shown in Supplementary Table 1c. (a) Alignment of all H5MBCS HA CS sequences shown in Supplementary Figure 4. The asterisk indicates

the border between HA1 and HA2. Results from testing (b) H5$_{MBCS_A1059C}$ HA$_{SND}$s, (c) H5$_{MBCS_A1062G}$ HA$_{SND}$s and (d) H5$_{MBCS_A1059C_A1062C}$ HA$_{SND}$s.

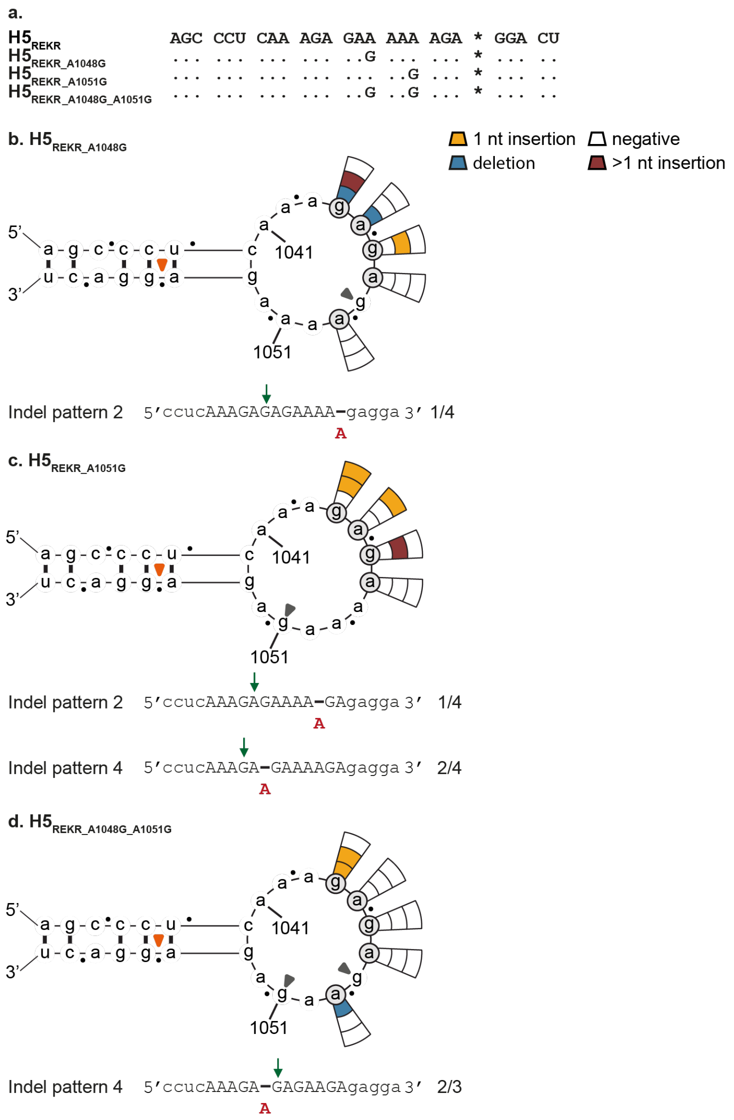

Supplementary Figure 5. Fewer indels were observed upon decreasing the A-stretch length at the 3' end of the H5$_{REKR}$ loop. Results are shown as described in the legend of Figure 1. Grey closed arrow heads refer to the introduced nucleotide substitutions as compared to H5$_{REKR}$. Observed indel patterns and corresponding frequencies are indicated below the structures, the other detected indels in are shown in Supplementary Table 1c. (a) Alignment of all H5$_{REKR}$ HA CS sequences shown in Supplementary Figure 5. The asterisk indicates the border between HA1 and HA2. Results from testing (g) H5$_{REKR_A1048G}$ HA$_{SND}$s, (c) H5$_{REKR_A1051G}$ HA$_{SND}$s and (d) H5$_{REKR_A1048G_A1051G}$ HA$_{SND}$s.

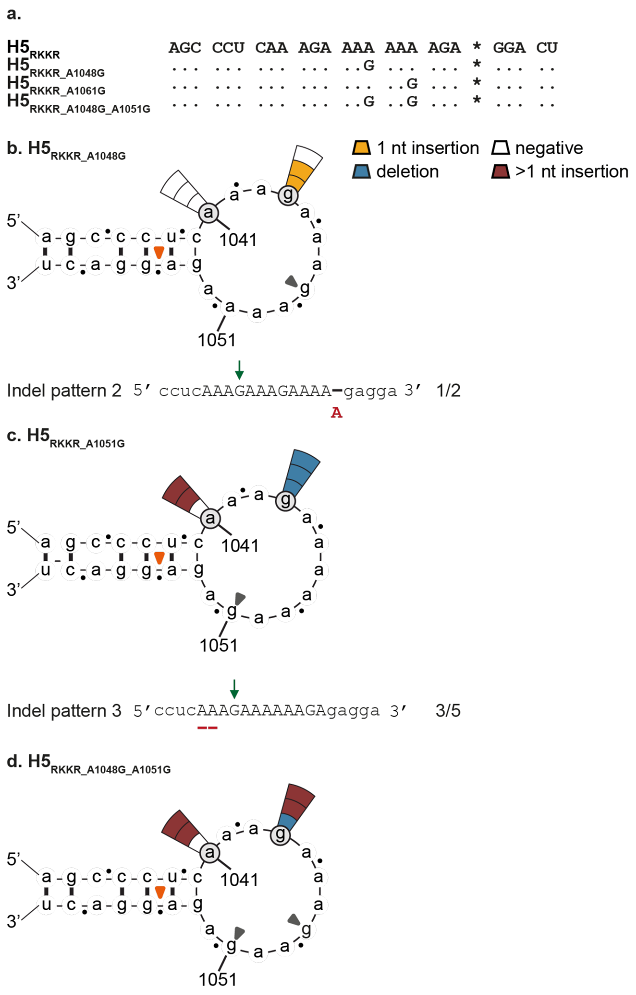

Supplementary Figure 6. Indel detection upon decreasing the A-stretch length at the 3' end of the H5RKKR loop. Results are shown as described in the legend of Figure 1. Grey closed arrow heads refer to the introduced nucleotide substitutions as compared to H5RKKR. Observed indel patterns and corresponding frequencies are indicated below the structures, the other detected indels are shown in Supplementary Table 1c. (a) Alignment of all H5RKKR HA CS sequences shown in Supplementary Figure 6. The asterisk indicates the border between HA1 and HA2. Results from testing (b) H5RKKR_A1048G HASNDs, (c) H5RKKR_A1051G HASNDs and (d) H5RKKR_A1048G_1051G HASNDs.

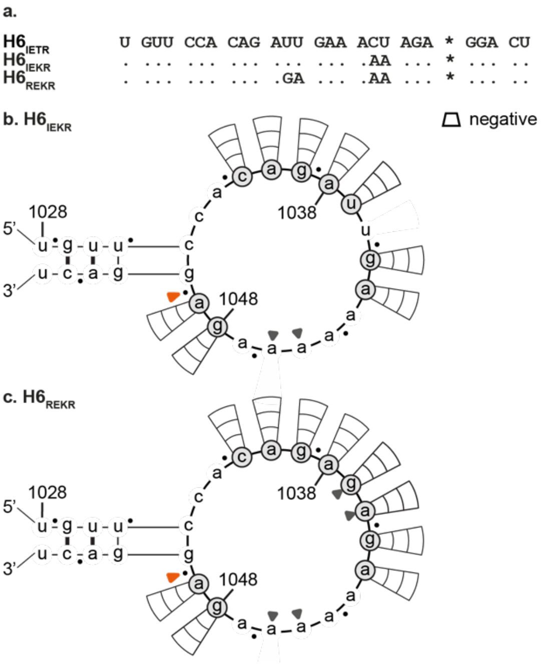

Supplementary Figure 7. No indels were observed upon using $H6_{IETR}$ and $H6_{REKR}$ HA_{SNDS}. Results are shown as described in the legend of Figure 1. Grey closed arrow heads refer to the introduced nucleotide substitutions as compared to $H6_{IETR}$. (a) Alignment of $H6_{IEKR}$ and $H6_{REKR}$ HA CS sequences shown in Supplementary Figure 7. The asterisk indicates the border between HA1 and HA2. Results from testing (b) $H6_{IEKR}$ HA_{SND}s and (c) $H6_{REKR}$ HA_{SND}s.

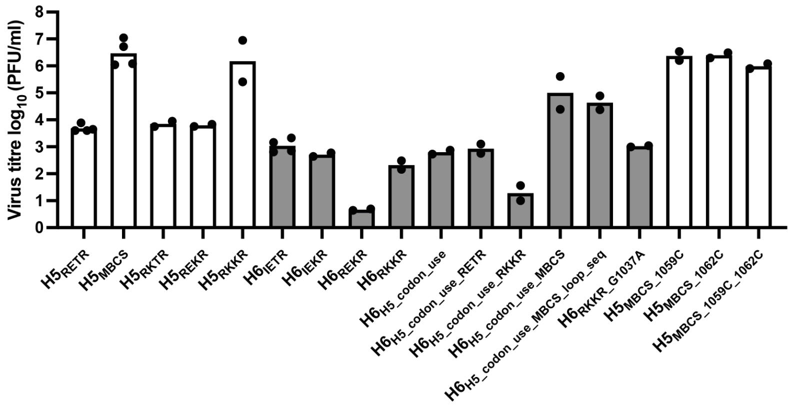

Supplementary Figure 8. Virus titres of viruses carrying the different HAs used in this study. All WT HAs that were used as templates to produce HA_{SND}s, except those with silent substitutions, were tested in a plaque assay using 293T supernatant. Virus titres are expressed as log_{10} plaque forming units (PFU)/ml. Dots correspond to individual data points and bars to the mean of independent reverse genetics and plaque assay experiments. $H5_{RETR}$, $H5_{MBCS}$ and $H6_{IETR}$ viruses were included as internal controls between experiments and showed comparable titres ($H5_{RETR}$ mean log_{10} (PFU/ml) = 3.69, SD = 0.14; $H5_{MBCS}$ mean log_{10} (PFU/ml) = 6.47, SD = 0.49; and $H6_{IETR}$ mean log_{10} (PFU/ml) = 3.04, SD = 0.49). Data from H5 and H6 viruses are represented by white and grey bars, respectively.

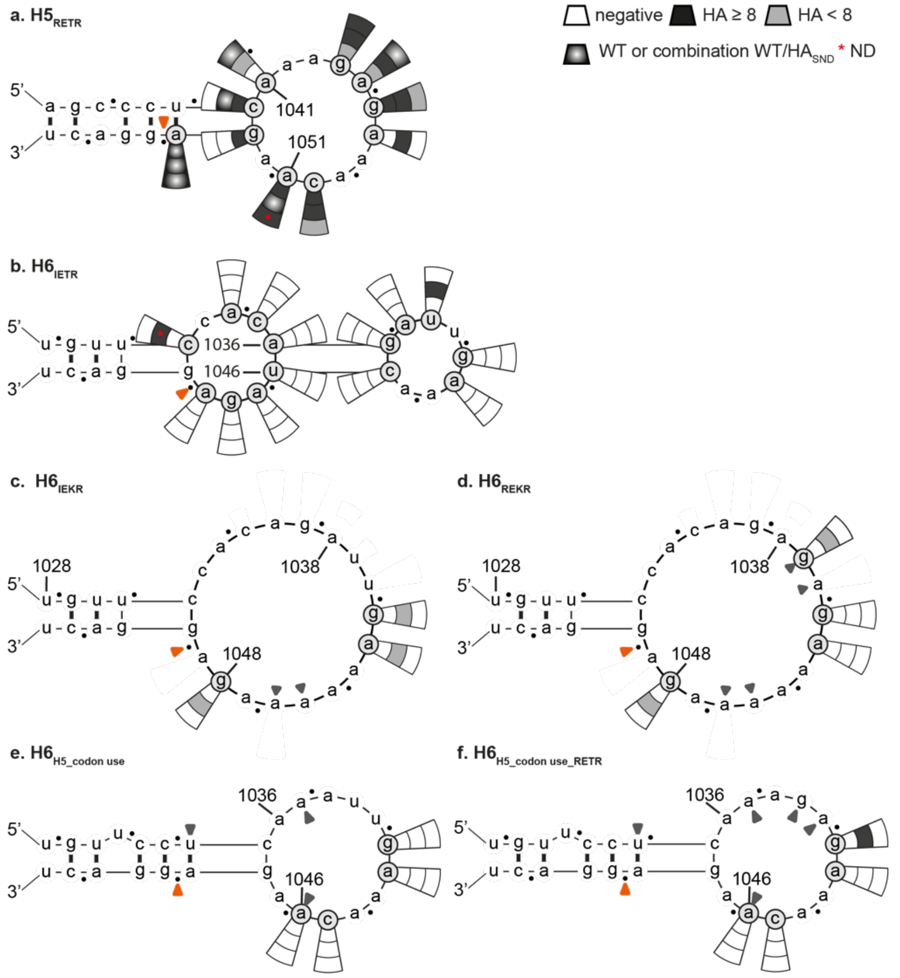

Supplementary Fig. 9. *Trans*-complementation with H5$_{RETR}$ HA protein led to increased indel detection in H5, but not in H6.

pCAGGS H5$_{RETR}$ expression plasmid was co-transfected during reverse genetics. HA CS PCR fragments from all samples with an HA titre of ≥8 HAU/25 µl were subsequently subjected to TOPO-cloning and the sequences of the corresponding 6 clones are indicated in Supplementary Table 1b. A white wedge indicates that no infectious virus was detected, a grey wedge indicates that the HA titre of the virus containing supernatant was ≤8 HAU/25 µl, a black wedge indicates an HA titre of ≥8 HAU/25 µl and a gradient wedge indicates WT or a combination of WT and SND sequences. A red asterisk indicates that sequencing of TOPO-clones was unsuccessful. Results from testing (a) H5$_{RETR}$ HA$_{SND}$s, (b) H6$_{IETR}$ HA$_{SND}$s, (c) H6$_{IEKR}$ HA$_{SND}$s, (d) H6$_{REKR}$ HA$_{SND}$s, (e) H6$_{H5_codon\ use}$ HA$_{SND}$s and (f) H6$_{H5_codon\ use_RETR}$ HA$_{SND}$s.

References

1. Gultyaev AP, Richard M, Spronken MI, Olsthoorn RCL, Fouchier RAM. Conserved structural RNA domains in regions coding for cleavage site motifs in hemagglutinin genes of influenza viruses. *Virus Evol* **5**, vez034 (2019).

2. Gultyaev AP, Spronken MI, Richard M, Schrauwen EJ, Olsthoorn RC, Fouchier RA. Subtype-specific structural constraints in the evolution of influenza A virus hemagglutinin genes. *Sci Rep* **6**, 38892 (2016).

3. Obenauer JC*, et al.* Large-scale sequence analysis of avian influenza isolates. *Science* **311**, 1576-1580 (2006).

4. Garcia M*, et al.* Evolution of H5 subtype avian influenza A viruses in North America. *Virus Res* **51**, 115-124 (1997).
